# Site and duration of abdominal pain discriminate symptomatic uncomplicated diverticular disease from previous diverticulitis patients

**DOI:** 10.1007/s11739-024-03588-6

**Published:** 2024-04-27

**Authors:** Marilia Carabotti, Giovanni Marasco, Caterina Sbarigia, Rosario Cuomo, Giovanni Barbara, Fabio Pace, Giovanni Sarnelli, Bruno Annibale, at behalf of REMAD group, Alida Andrealli, Sandro Ardizzone, Marco Astegiano, Francesco Bachetti, Simona Bartolozzi, Stefano Bargiggia, Gabrio Bassotti, Maria Antonia Bianco, Giuseppe Biscaglia, Matteo Bosani, Maria Erminia Bottiglieri, Martina Cargiolli, Carolina Ciacci, Antonio Colecchia, Agostino Di Ciaula, Alessandra Dell’Era, Marina De Matthaeis, Mirko Di Ruscio, Marco Dinelli, Virginia Festa, Ermenegildo Galliani, Bastianello Germanà, Mario Grassini, Ennio Guido, Franco Iafrate, Paola Iovino, Donato Iuliano, Andrea Laghi, Giovanni Latella, Gianpiero Manes, Elisa Marabotto, Alessandro Moscatelli, Riccardo Nascimbeni, Pietro Occhipinti, Marco Parravicini, Marco Pennazio, Sergio Peralta, Piero Portincasa, Franco Radaelli, Raffaella Reati, Alessandro Redaelli, Marco Rossi, Raffale Salerno, Sergio Segato, Carola Severi, Giuseppe Scaccianoce, Valentina Valle, Clara Virgilio, Angelo Viscido

**Affiliations:** 1 Department of Medical-Surgical Sciences and Translational Medicine, Sapienza University, Via di Grottarossa 1035-1039, 00189 Rome, Italy; 2 Department of Medical and Surgical Sciences, University of Bologna, 40138 Bologna, Italy; 3 UOC of Gastroenterology, AORN Sant’Anna e San Sebastiano, 81100 Caserta, Italy; 4 UOC of Gastroenterology, Bolognini Hospital, 24068 Seriate, Italy; 5 Department of Clinical Medicine and Surgery, University of Naples Federico II, 80131 Naples, Italy

**Keywords:** Abdominal pain, Diverticular diseases, Diverticulitis, Irritable bowel syndrome, Questionnaires

## Abstract

Abdominal pain in patients with diverticular disease (DD) can be challenging in clinical practice. Patients with symptomatic uncomplicated diverticular disease (SUDD) and patients with a previous acute diverticulitis (PD) may share a similar clinical pattern, difficult to differentiate from irritable bowel syndrome (IBS). We used standardized questionnaires for DD (short and long lasting abdominal pain) and IBS (following Rome III Criteria) to assess clinical features of abdominal pain, in terms of presence, severity and length, in SUDD and PD patients. One hundred and forty-eight SUDD and 118 PD patients completed all questionnaires. Short-lasting pain was more frequent in SUDD than PD patients (*p* = 0.007). Number of long-lasting pain episodes was higher in SUDD (6.6 ± 11.9) compared to PD patients (3.4 ± 6.9) (*p* < 0.001). PD patients reported long-lasting pain more frequently in the lower left abdomen (*p* < 0.001), while in SUDD it was more frequently diffuse (*p* = 0.002) or localized in the lower right quadrant (*p* = 0.009). Features associated with long-lasting pain (fever, confinement to bed, consultations, antibiotic therapy, hospitalization) were more often reported in PD patients. IBS criteria were reported in 28.2% of patients and were more frequent in SUDD than PD patients (37.2% vs 17.1%, *p* < 0.001). SUDD and PD patients presented different pattern of abdominal pain (length, number of long lasting episodes, site and associated features), with a third reporting overlap with IBS. Further observational studies are needed to better characterize abdominal symptoms in DD patients, especially in those not fulfilling IBS criteria.

*Trial registration*: The REMAD Registry is registered as an observational study in ClinicalTrial.gov (ID: NCT03325829).

**Supplementary Information:**

The online version contains supplementary material available at 10.1007/s11739-024-03588-6.

## Introduction

Colonic diverticula are a frequent condition in Western countries, which prevalence increases with age, affecting up to two-thirds of people older than 80 years [1]. Most patients will remain asymptomatic throughout their lifetime (diverticulosis), whereas about one fifth may develop chronic abdominal symptoms including abdominal pain, bloating and changes in bowel habits, a condition termed symptomatic uncomplicated diverticular disease (SUDD). A minority of patients, about 1–4%, may develop acute diverticulitis, an inflammatory condition that may evolve in complications like abscesses, perforation, fistula, obstruction, or peritonitis [2]. Patients with a previous diverticulitis (PD) may experience recurrent diverticulitis in about 20% of cases and/or complain chronic-recurrent gastrointestinal symptoms in absence of inflammation [3, 4]. Simpson et al. show that about 67% of patients hospitalized for acute diverticulitis developed new, recurrent, short-lived abdominal pain following discharge [4]. Similarly, Cohen E et al. in a retrospective cohort study show that subjects with previous acute diverticulitis are 4.7 times more at risk for developing irritable bowel syndrome (IBS) than controls, configuring a condition called “post-diverticulitis IBS” [5].

Patients with symptomatic diverticular disease (SUDD and PD) may share a similar clinical pattern characterized by chronic-recurrent symptoms like abdominal pain, bloating and changes of bowel habits, difficult to differentiate from IBS. Symptoms attributable to diverticula overlapped with IBS in 6–60% of cases [6, 7] but it has been reported that some clinical characteristics may help in differentiating these conditions [8, 9]. For these reasons, an accurate characterization of abdominal pain in patients with diverticular disease (DD) can be very challenging in clinical practice.

Aim of this study was to assess the clinical features of abdominal pain, in terms of presence, severity and length, in patients with SUDD and PD using standardized questionnaires for DD and IBS.

## Materials and methods

### Study design

We utilized the REMAD registry, a 5-year, prospective, observational, multicentre, cohort study in which, a total of 1217 patients from 47 Italian centres were enrolled during the 2 months recruitment period and followed up for 5 years (from April 2015 to April 2020). Materials and methods were reported in previous papers [10–12]. Briefly, inclusion criteria were: informed consent; age $\ge$ 18 years; endoscopic/radiological-confirmed colonic diverticula. Exclusion criteria were: failure to sign informed consent; inability to adhere to the study procedures.

### Data collection

At entry, patients were categorized according to the following criteria: (i) diverticulosis, presence of diverticula in the absence of abdominal symptoms; (ii) SUDD, recurrent abdominal pain mainly in the lower abdominal quadrants, with a frequency of at least once a week, present for at least 6 months, and/or changes in bowel habit, without a well-defined previous attack of acute diverticulitis; (iii) PD, patients who experienced at least one episode of acute diverticulitis, complicated or not; when available medical charts were reviewed [10, 11]. Demographic and personal data, life-styles factors and assumption of drugs with gastrointestinal (GI) effect were collected. Patients who fulfilled criteria for diagnosis of SUDD and PD were asked to fill in questionnaires for characterization of abdominal pain: (i) questionnaire for short-lasting pain (< 24 h); (ii) questionnaire for long-lasting pain (> 24 h); (iii) questionnaire for irritable bowel syndrome (IBS) following Rome III Criteria. Patients with diverticulosis were not invited to complete the questionnaires as they were asymptomatic and were excluded from the current analysis. Questionnaires were administrated during outpatient’s visits, face to face, by dedicated physicians.

### Abdominal short-lasting pain questionnaire

Short-lasting pain was defined as presence of episodes of abdominal pain lasting lesser than 24 h in the last 6 months. The questionnaire assessed: (i) pain’s abdominal localization (upper left, lower left, upper right, lower right and diffuse), (ii) the association with bowel habits (pain improved/not improved/partially improved by defecation), (iii) pain’s severity by means of a visual analogue scale (VAS) and semi quantitatively as mild (not influencing usual activities/causing minimal discomfort), moderate (clearly present and bothersome) or severe (not allowing normal daily activities) [4, 6, 13]. For abdominal pain localization, more than one abdomen section could be selected.

### Abdominal long-lasting pain questionnaire

Long-lasting pain was defined as presence of episodes of abdominal pain persisting more than 24 h in the last 6 months. This questionnaire evaluated the presence of: (i) number of episodes of abdominal pain, (ii) abdominal pain’s localization (upper left, lower left, upper right, lower right and diffuse), (iii) if pain improves with the use of antispasmodics, (iv) severity of pain both by means of a VAS and semi quantitatively as mild (not influencing usual activities/causing minimal discomfort), moderate (clearly present and bothersome) or severe (not allowing normal daily activities). Moreover, some characteristics associated to long-lasting pain were investigated: presence of fever, confinement to bed, medical consultation, antibiotic therapy and hospital admission [4, 6, 13].

### Questionnaire for irritable bowel syndrome

The diagnosis of IBS was based on the following criteria according to Rome III: presence of abdominal discomfort or pain at least 3 days/month in the last 3 months, with onset at least 6 months prior to diagnosis, associated with two or more of the following three characteristics: (i) relief with defecation, and/or (ii) onset associated with a change in frequency of stool, and/or (iii) onset associated with a change in aspect of stools [14]. IBS subtypes based on bowel habits: IBS-D (diarrhea predominant), IBS-C (constipation predominant), IBS-M (mixed predominant, alternating bowel habits), and IBS-U (unclassifiable bowel habits) were determined with the aid of the Bristol Stool Form Scale [15].

### Statistical analysis

All data were first tested with the Shapiro Wilk test for checking normal distribution, and after were presented as counts and percentages when categorical variables and mean and standard deviation (SD) when continuous variables. The categorical variables were compared using the Chi-squared or Fisher’s exact tests as appropriate. For multiple categorical variables, the Chi-squared test of independence was used. The continuous variables were compared using the *t* test. The differences in demographics and clinical characteristics of patients with SUDD and PD cohorts were calculated. Subsequently, factors associated with SUDD versus PD diagnosis were explored using univariate and multivariate analysis. The estimated odds ratios (OR) with their 95% confidence intervals (CI) were calculated. The probability values were two-sided; a probability value of less than 0.05 was considered statistically significant. Statistical analysis was performed with STATA 17.0 (SE, Standard Edition, College Station, TX: StataCorp LP).

## Results

**Fig.1:**
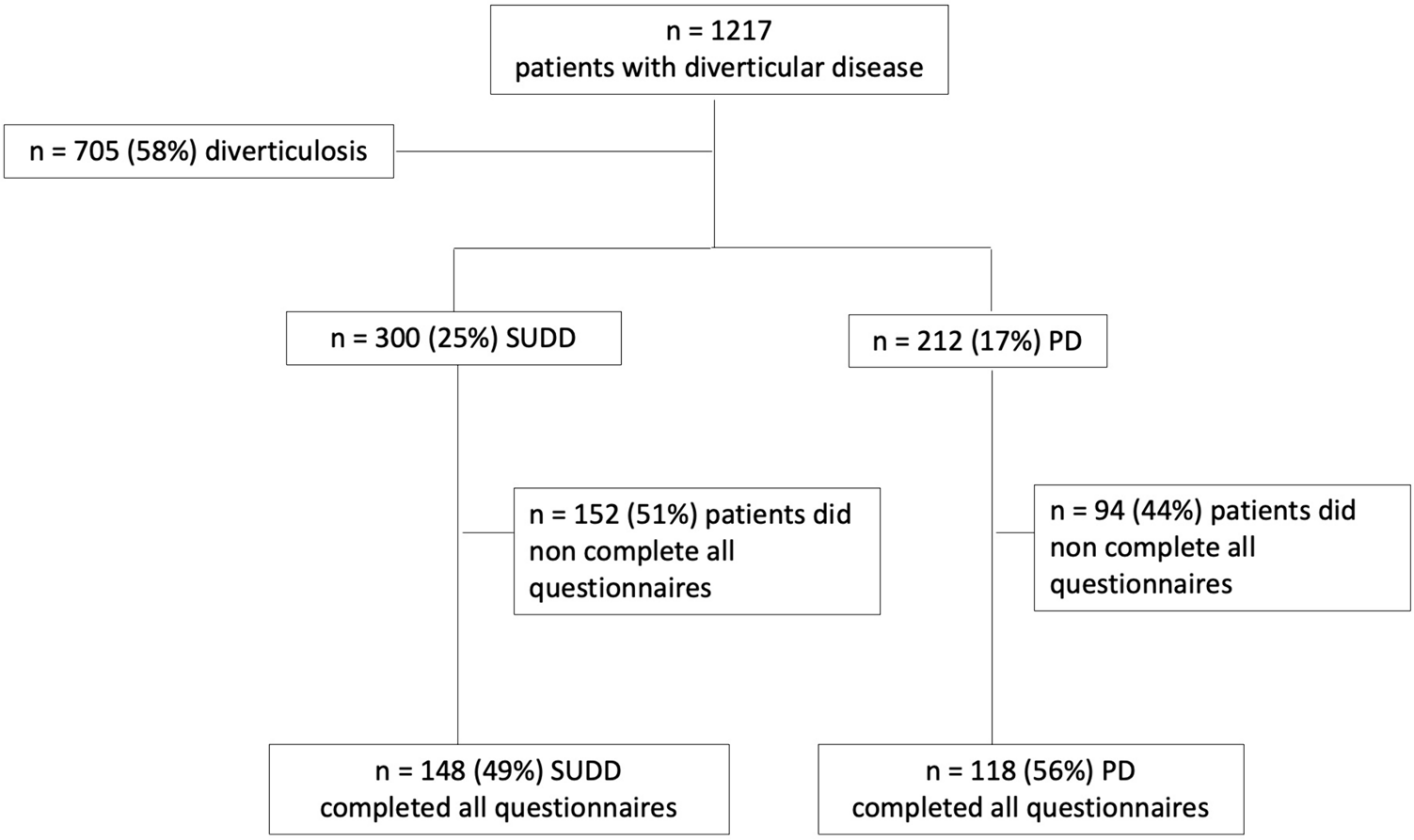
Flowchart of the study population. *SUDD* symptomatic uncomplicated diverticular disease, *PD* previous diverticulitis

From the 1217 patients included in the REMAD registry, 705 patients (57.9%) fulfilled criteria for diagnosis of diverticulosis, 300 patients (24.7%) fulfilled criteria for SUDD and the remaining 212 patients (17.4%) fulfilled criteria for PD. Patients with diverticulosis (*n* = 705) were excluded from the current analysis. Among them, 148 out of 300 patients with SUDD and 118 out of 212 patients with PD completed all questionnaires, representing the study population (Fig. 1). In PD patients, diverticulitis was diagnosed in 78.9% with abdominal computed tomography and in 21.1% with abdominal ultrasound. 96.6% of patients with acute diverticulitis have available medical charts, whereas in 3.4% of patients the diagnosis was based on clinical and biochemical criteria by expert physicians. Differences regarding patient’s demographic and clinical characteristics between the SUDD and PD patients are reported in Table 1. Age was significantly lower in PD compared to SUDD patients (*p* = 0.027). There were no significant differences between patients in terms of gender and BMI. Regarding the main reason for DD diagnosis, abdominal pain was significantly more frequent in PD (*p* = 0.003), while bloating was only present in SUDD compared to PD patients (4.7% vs 0%, *p* = 0.019). Regarding lifestyle factors, active smokers were more frequent in PD compared to SUDD patients (*p* = 0.031), but no significant differences were found in terms of alcohol, coffee intake and physical activity.

**Table 1:** Demographic and clinical characteristics of symptomatic uncomplicated diverticular disease and previous diverticulitis patients

	SUDD (*n* = 148)	PD (*n* = 118)	*p* value
Gender			
Females (%)	85 (57.4)	62 (52.5)	0.426
Age (years), mean ± SD	66.1 ± 10.2	62.7 ± 12.9	**0.027**
BMI (kg/m^2^), mean ± SD	26.4 ± 3.8	26.1 ± 4.8	0.222
Main reason for DD diagnosis			
Abdominal pain (%)	93 (62.8)	94 (79.7)	**0.003**
Alteration of bowel habits (%)	12 (8.1)	4 (3.4)	0.108
Rectal bleeding (%)	7 (4.7)	10 (8.5)	0.215
Bloating (%)	7 (4.7)	0 (0.0)	**0.019**
Anemia (%)	2 (1.4)	1 (0.9)	0.699
Fever (%)	0 (0.0)	3 (2.5)	0.086
Lifestyle factors			
Active Smokers (%)	17 (11.5)	25 (21.1)	**0.031**
Number cigarettes/day	20.2 (± 16)	14.3 (± 10)	0.079
Years smoking	26.4 (± 11.7)	24.8 (± 12.9)	0.419
Use of Alcohol (%)	50 (33.8)	54 (45.8)	0.073
Alcoholic unit/day	12.5 (± 46.3)	4.3 (± 17)	0.758
Use of Coffee (%)	114 (77.0)	102 (86.4)	0.097
Cups/day	2.3 (± 1.5)	2.1 (± 1.3)	0.439
Practice physical activity (%)	55 (37.2)	48 (40.7)	0.396
Hours/week	4.7 (± 4.1)	4 (± 2.5)	0.706
Drugs with GI effect (in the last 12 months)			
NSAIDs	45 (30.4)	27 (22.9)	0.170
PPI	61 (41.2)	43 (36.4)	0.726
Rifaximin	53 (68.8)	39 (55.7)	0.101
Mesalazine	20 (13.7)	37 (31.4)	**0.001**
Probiotics	31 (21.2)	26 (22)	0.875
Prebiotics	10 (6.9)	5 (4.2)	0.362
Antispasmodics	6 (4.1)	6 (5.1)	0.705

Data reported in bold are statistically significant

*BMI* Body Mass Index, *DD* Diverticular Disease, *GI* gastrointestinal, *NSAIDs* Non-steroidal anti-inflammatory drugs, *PD* Previous Diverticulitis, *PPI* Proton pump inhibitors, *SUDD* Symptomatic Uncomplicated Diverticular Disease

Except for the use of mesalazine that was more frequent in PD patients (*p* = 0.001), no differences regarding the use of drugs with GI effect assumed in the previous 12 months was found.

### Pattern of short-lasting and long-lasting abdominal pain

**Fig. 2:**
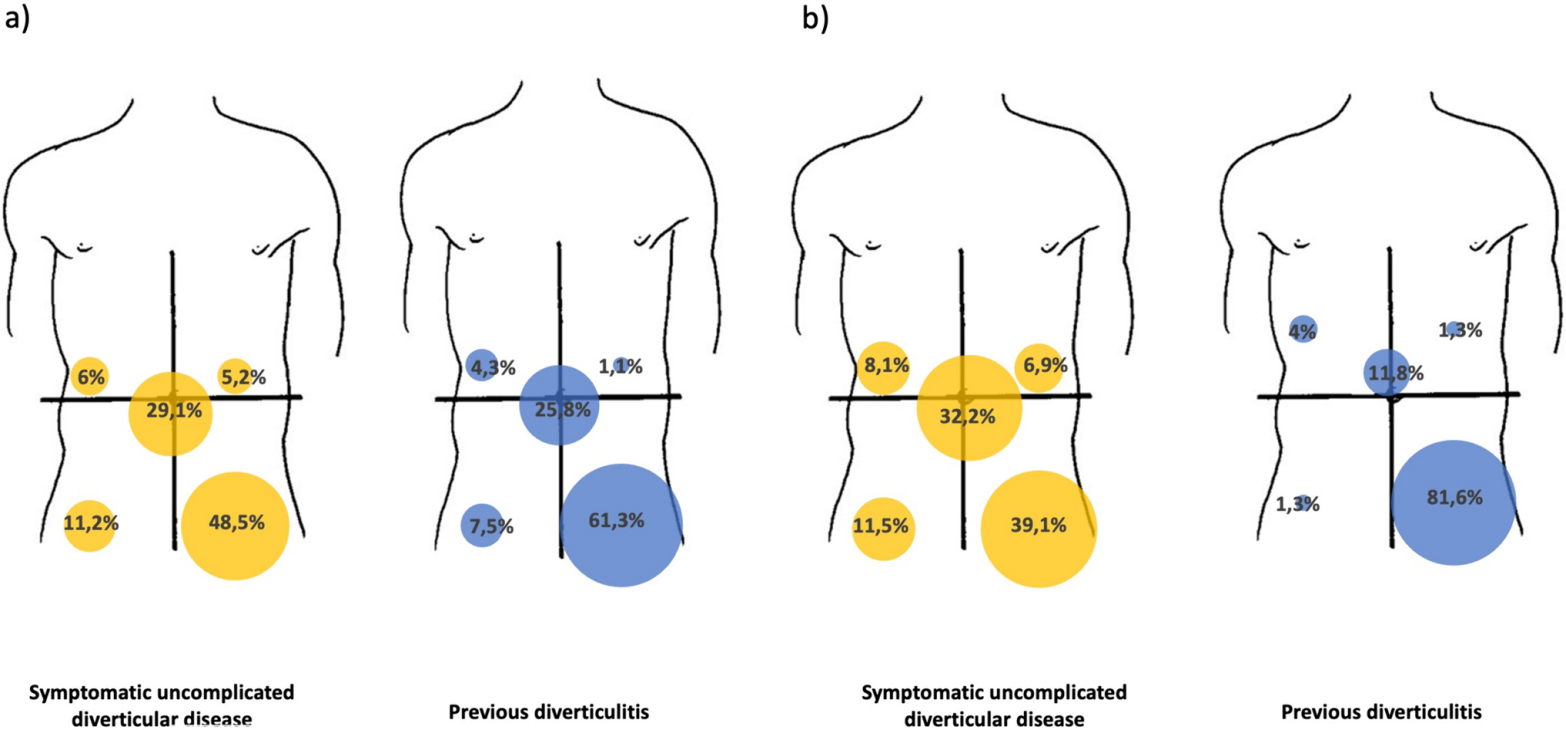
Size and prevalence of short (**a**) and long lasting (**b**) abdominal pain localization in symptomatic uncomplicated diverticular disease and previous diverticulitis patients

Table 2 shows the characteristics of short-lasting abdominal pain in SUDD and PD patients. Episodes of short-lasting abdominal pain, although highly prevalent in both groups, were significantly more frequently in SUDD than in PD patients (*p* = 0.007). No significant differences in terms of abdominal pain’s severity (by means of a VAS scale and semi quantitatively) or pain’s relief with defecation were found. Upper left abdomen localization was poorly observed, although more frequent in SUDD patients than PD (5.2% vs 1.1%, *p* < 0.001) (Fig. 2a).

**Table 2:** Characteristics of short-lasting abdominal pain (< 24 h)

	SUDD (*n* = 148)	PD (*n* = 118)	*p* value
Presence of short-lasting abdominal pain in the last 6 months (%)	134 (90.5)	93 (78.8)	**0.007**
Localization^a^			
Lower right abdomen (%)	15 (11.2)	7 (7.5)	0.358
Lower left abdomen (%)	65 (48.5)	57 (61.3)	0.057
Upper right abdomen (%)	8 (6)	4 (4.3)	0.581
Upper left abdomen (%)	7 (5.2)	1 (1.1)	** < 0.001**
Diffuse (%)	39 (29.1)	24 (25.8)	0.585
Pain disappearing after defecation^a^			
Yes (%)	55 (41)	33 (35.5)	0.398
No (%)	28 (20.9)	29 (31.2)	0.079
Not Always (%)	50 (37.3)	31 (33.3)	0.538
VAS (mm; mean ± SD)	5.1 ± 2.2	5.4 ± 2.4	0.308
Severity^a^			
Mild (%)	45 (33.6)	30 (32.3)	0.835
Moderate (%)	79 (59)	50 (53.8)	0.437
Severe (%)	10 (7.5)	13 (14)	0.110

Data reported in bold are statistically significant

*PD* Previous Diverticulitis, *SUDD* Symptomatic Uncomplicated Diverticular Disease, *VAS* Visual Analogue Scale

^a^Data are reported on patients having short-lasting abdominal pain

Regarding long-lasting abdominal pain, differences between SUDD and PD groups are reported in Table 3. Presence of episodes of long-lasting abdominal pain was similar between SUDD and PD (59% vs 64%) but the number of episodes was significatively higher in SUDD (6.6 ± 11.9) compared to PD patients (3.4 ± 6.9) (*p* < 0.001). In terms of pain localization, PD patients reported long-lasting pain more frequently in the lower left abdomen (*p* < 0.001), while in SUDD it was more frequently diffuse (*p* = 0.002) or localized in the lower right quadrant (*p* = 0.009) (Fig. 2b). Moreover, features associated with long-lasting abdominal pain (fever, confinement to bed, consultations, antibiotic therapy, and hospitalization) were significantly more often reported in patients with PD than in patients with SUDD (Table 3). In patients using antispasmodics, pain improvement was similar between the two groups of patients.

**Table 3:** Characteristics of long-lasting abdominal pain (> 24 h)

	SUDD (*n* = 148)	PD (*n* = 118)	*p* value
Presence of long-lasting abdominal pain in the last 6 months (%)	87 (58.8)	76 (64.4)	0.281
Number of episodes in the last 6 months (mean ± SD)	6.6** ± **11.9	3.4** ± **6.9	** < 0.001**
Localization^a^			
Lower right abdomen (%)	10 (11.5)	1 (1.3)	**0.009**
Lower left abdomen (%)	34 (39.1)	62 (81.6)	** < 0.001**
Upper right abdomen (%)	7 (8.1)	3 (4)	0.277
Upper left abdomen (%)	6 (6.9)	1 (1.3)	0.080
Diffuse (%)	28 (32.2)	9 (11.8)	**0.002**
Severity^a^			
Mild (%)	19 (21.8)	8 (10.5)	0.104
Moderate (%)	47 (54.0)	42 (55.2)	1.00
Severe (%)	21 (24.1)	26 (34.2)	0.169
VAS (mm), mean ± SD	5.8 ± 2.5	6.3 ± 2.6	0.117
Features associated with abdominal pain^a^			
Fever (%)	8 (9.2)	32 (42.1)	** < 0.001**
Confinement to bed (%)	26 (29.9)	38 (50)	**0.009**
Consultations (%)	35 (40.2)	46 (60.5)	**0.010**
Antibiotic therapy (%)	30 (34.5)	50 (65.8)	** < 0.001**
Hospitalization (%)	12 (13.8)	34 (44.7)	** < 0.001**
Is there an improvement in patients using antispasmodics?^a^			
Yes (%)	28 (32.2)	22 (29)	** 0.655**
No (%)	18 (20.7)	19 (25)	0.512
Not always (%)	18 (20.7)	20 (26.3)	0.397

Data reported in bold are statistically significant

*PD* Previous Diverticulitis, *SUDD* Symptomatic Uncomplicated Diverticular Disease, *VAS* Visual Analogue Scale

^a^Data are reported on patients having long-lasting abdominal pain

Finally, considering together SUDD and PD patients, 132 out of 266 patients (49.6%) reported an overlap of short- and long-lasting abdominal pain, without significant differences between SUDD (*n* = 72, 54.5%) and PD (*n* = 60, 45.5%) (*p* = 0.805).

Univariate and multivariate analyses assessing pain-associated factors independently associated with PD diagnosis were performed as a post-hoc analysis (Table 4). At univariate analysis, we found a significant inverse association of PD diagnosis with the presence of short-lasting abdominal pain and the number of episodes of long lasting abdominal pain, while we found a positive correlation with lower left long lasting abdominal pain and its severity. At multivariate analysis only the lower left pain localization for long-lasting abdominal pain resisted as a predictive factor for PD diagnosis.

**Table 4:** Univariate and multivariate analysis evaluating pain characteristics associated with PD diagnosis

	Univariate analysis		Multivariate analysis	
	OR (95% CI)	*p* value	OR (95% CI)	*p* value
Presence of short-lasting abdominal pain in the last 6 months (%)	0.306 (0.154–0.610)	**0.001**		
Localization of short lasting abdominal pain^a^				
Lower right abdomen (%)	Referent	Referent		
Lower left abdomen (%)	1.879 (0.716–4.932)	0.2		
Upper right abdomen (%)	1.071 (0.239–4.794)	0.928		
Upper left abdomen (%)	0.306 (0.031–2.991)	0.309		
Diffuse (%)	1.319 (0.470–3.698)	0.599		
Short lasting abdominal pain disappearing after defecation^a^	1.007 (0.740–1.370)	0.966		
VAS Short lasting abdominal pain	1.052 (0.934–1.186)	0.398		
Severity of Short lasting abdominal pain^a^	1.230 (0.799–1.892)	0.346		
Presence of long-lasting abdominal pain in the last 6 months (%)	1.367 (0.837–2.234)	0.212		
Number of episodes of long-lasting abdominal pain in the last 6 months (mean ± SD)	0.953 (0.905–1.005)	**0.077**		
Localization of long-lasting abdominal pain^a^				
Lower right abdomen (%)	Referent	Referent	Referent	Referent
Lower left abdomen (%)	18.235 (2.238–148.577)	**0.007**	14.154 (1.654–121.116)	**0.016**
Upper right abdomen (%)	4.286 (0.366–50.197)	0.246		
Upper left abdomen (%)	1.668 (0.087–31.869)	0.734		
Diffuse (%)	3.214 (0.360–28.678)	0.296		
Severity of long-lasting abdominal pain^a^	1.683 (1.038–2.728)	**0.035**		
VAS (mm) of long-lasting abdominal pain, mean ± SD	1.083 (0.957–1.227)	0.207		
Improvement in patients using antispasmodics^a^	1.194 (0.783–1.822)	0.408		

Data reported in bold are statistically significant

*PD* Previous Diverticulitis, *SUDD* Symptomatic Uncomplicated Diverticular Disease, *VAS* Visual Analogue Scale

^a^Data are reported on patients having long-lasting abdominal pain

### Overlap with irritable bowel syndrome

**Fig. 3:**
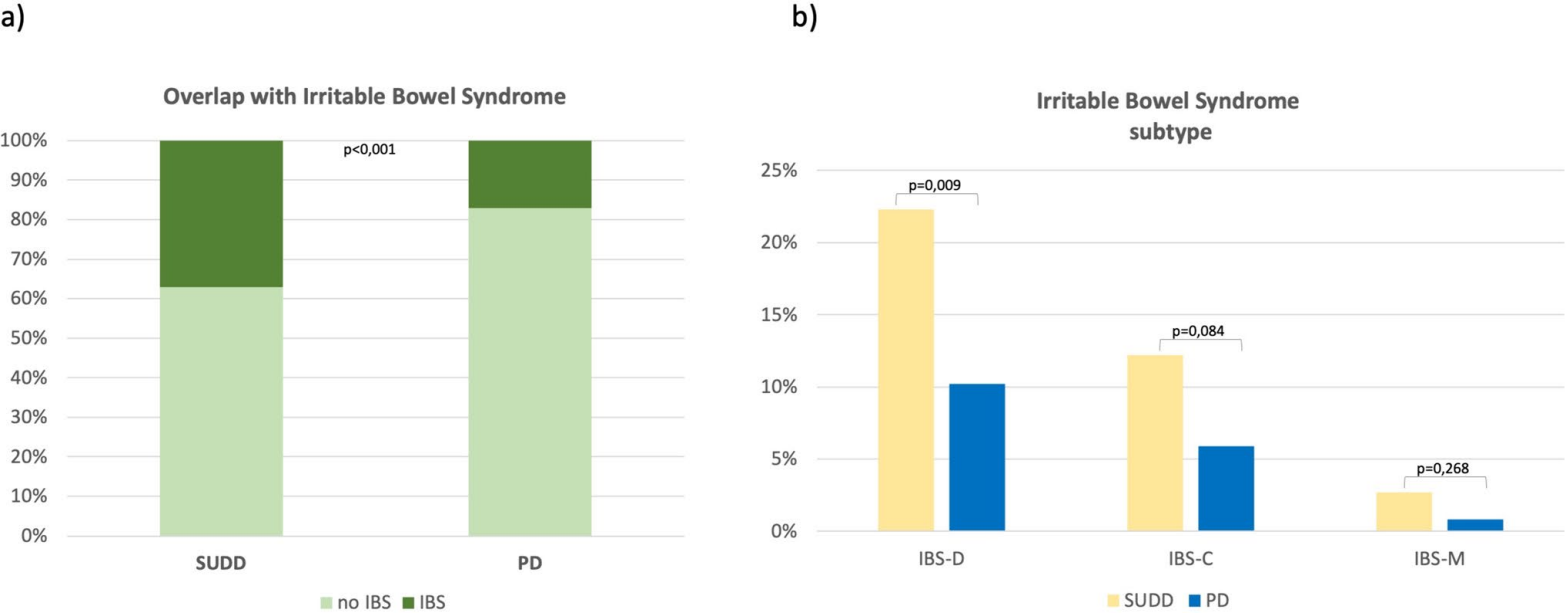
Overlap between irritable bowel syndrome in patients with symptomatic uncomplicated diverticular disease and previous diverticulitis (**a**) and irritable bowel syndrome subtypes prevalence (**b**)

Diagnosis of IBS according to Rome III Criteria was reported in 75 patients (28.2%) and was more frequent in SUDD patients than in PD (37.2% vs 17.1%, *p* < 0.001) (Fig. 3a). Patients fulfilling the criteria for IBS diagnosis were stratified according to the bowel habits (IBS with diarrhoea, IBS with constipation, IBS mixed) (Fig. 3b). The most prevalent IBS subtype was the IBS-D, that was present in the 45 patients out of 75 with IBS (61.3%) [SUDD 33 (22.3%) vs. PD 12 (10.2%), *p* = 0.009], whereas IBS-C was found in 25 patients (33.3%) [SUDD 18 (12.2%) vs. PD 7 (5.9%), *p* = 0.084] and IBS-M in 5 patients (5.3%) [SUDD 4 (2.7%) vs. 1 (0.8%), *p* = 0.268].

## Discussion

This observational multicentre cross-sectional study aimed to characterize abdominal pain in patients with symptomatic diverticular disease using standardized questionnaire for DD and IBS. In fact, it is matter of debate whether SUDD could be considered a disease its own or it represents the coexistence of IBS in patients with colonic diverticula; furthermore, after an episode of acute diverticulitis, patients may complain of chronic-recurrent abdominal pain, only sometimes related to a “low grade” recurrent diverticulitis [5].

Regarding short-lasting abdominal pain, we found that SUDD patients presented more often episodes of pain compared to PD patients, without significant differences in terms of pain severity or correlation of pain with defecation. At our knowledge, no study has directly compared SUDD and PD patients in terms of short-lasting abdominal pain. We found that about 90% of SUDD patients complained of short-lasting abdominal pain; this percentage seemed to be higher compared to 33–36% found, respectively, by Humes and Simpson [4, 16]. We believe that one of the main reason might be ascribed to the different way of questionnaires administration (postal in the cited two studies vs face-to-face interviews in our study) as recently reported [17]. However, previous studies from our group showed similar findings, with 70% and 82% of DD patients complaining of short-lasting abdominal pain [6, 13]. Regarding PD patients, Simpson et al. found that 69% of patients had short-lasting abdominal pain after a documented episode of acute diverticulitis, with a pain mean duration of 4 h and a pain intensity ranging from mild to moderate. This data might be considered in line with our findings, with 78% of PD patients complaining of short-lasting pain with a prevalence of moderate intensity in more than a third of patients (35.6%) [4].

Concerning the long-lasting abdominal pain (> 24 h), we found some interesting results. Although the proportion of SUDD and PD patients complaining of long-lasting pain was similar (58.8% vs 64.4%), the number of episodes was significantly higher in SUDD compared to PD. In current literature, the prevalence of long-lasting abdominal pain in patients with diverticular disease ranged from 5.6% to 100% [4, 6, 13, 16, 18] with no available data that directly compares SUDD and PD. We believe that there are at least two factors contributing to the wide observed range: first, some authors considered the presence of long-lasting pain as the main hallmark of SUDD diagnosis [18, 19] and others not [4, 6, 13]; second, often the population considered in these studies included patients with diverticulosis, potentially leading to misclassification bias. [4, 7, 16] We believe that considering as SUDD only patients presenting pain longer than 24 h may be limiting [20]; in fact, we demonstrated that more than 90% of SUDD patients complained of short-lasting abdominal pain, with about half of patients having also long-lasting pain. These data highlighted that abdominal pain regardless of its duration, is a remarkable symptom in this setting.

Moreover, we observed a different pattern of pain localization between SUDD and PD (Fig. 2a and b). In fact, we found that long-lasting abdominal pain was more frequently diffuse or localized in the lower right abdomen in SUDD patients than in PD (respectively, 32.2% vs 11.8%, *p* = 0.002 and 11.5% vs 1.3%, *p* = 0.009), while it was more frequently localized in the lower left abdomen in patients with PD (39.1% vs 81.6%, *p* < 0.001). Furthermore, at multivariate analysis only the lower left pain localization for long-lasting abdominal pain resisted as a predictive factor for PD diagnosis. Available data show that SUDD abdominal pain is predominantly localized in the lower left abdomen [8, 18, 21], while no studies assessed pain’s localization in PD patients. Furthermore, we showed that long-lasting abdominal pain was associated more frequently to occurrence of fever, confinement to bed, need for medical consultations, antibiotics therapies and hospitalization in patients with PD, than in SUDD. Considering together characteristics of short and long-lasting abdominal pain, these results suggested that SUDD complained symptoms more frequently than PD patients (i.e., higher occurrence of short lasting pain, higher number of episodes of long lasting pain), but with a less sever clinical phenotype (i.e., lower occurrence of fever, need of confinement to bed or need of antibiotic treatment), suggesting that in PD patients, chronic recurrent abdominal pain could sometimes underlying a possible recurrent diverticulitis. Another factor that might potentially contribute to this clinical scenario is smoking habit. Even if in the present study the number of active smokers is low, smokers are more frequent in PD compared to SUDD patients. A previous study showed that smoking was an independent factor associated with severe abdominal pain in IBS-M and IBS-C, regardless colonic transit time, supporting the hyphothesis that smoking might affect visceral perception and pain severity [22].

At now, even if the use of some biomarkers has been proposed for differential diagnosis between SUDD and IBS, such as fecal calprotectin [21, 23] or ultrasonography [24], no accepted criteria for SUDD diagnosis have been reached yet. In fact, only two small case–control studies, reported that fecal calprotectin levels (expressed semiquantitatively) are higher in SUDD compared to patients with IBS-like symptoms [21, 23], and no further larger studies are available. On the other hand, one study evaluated the role of intestinal ultrasound in discriminating SUDD among patients with abdominal symptoms. Patients with SUDD showed a significantly greater muscle thickness than those with IBS, patients with unclassifiable abdominal pain and healthy subjects, but comparable with those with diverticulosis [24]. Thus, available data are still scarce and then the clinical picture, particularly abdominal pain characteristics (length and pain localization), remained a reliable and largely used method to diagnose SUDD in clinical practice [6, 13, 21].

Chronic and recurrent abdominal symptoms associated to SUDD might be debilitating and might affect quality of life of patients [25]. In fact, some prospective studies have demonstrated an impairment in quality of life in SUDD patients compared to general population [26, 27]. Also, a more recent observational multicentre study has showed that quality of life in SUDD patients is similar to patients with a previous episode of diverticulitis, likely suggesting that the presence of troublesome recurrent abdominal symptoms is perceived a full disease similarly to patients who have experimented a diverticular complication [10].

Another interesting point is the current debate on the overlap between SUDD and IBS. In fact, some authors do not consider SUDD as a pathologic entity on its own, but merely the coexistence of IBS in patients with colonic diverticula [18, 21]. In our cohort we found that, according to Rome III Criteria, only about a third of SUDD patients fulfil criteria for IBS, suggesting that these patients can’t be all categorized as IBS, as two-thirds would remain outside this definition.

Another interesting finding in our study is the low occurrence of IBS-like symptoms in PD patients (17%), further suggesting that even after acute diverticulitis, which is a known predisposing condition, the overlap is weak. Previous data identified a wide overlap range between DD and IBS, from 6 to 60%, with high heterogeneity between studies, which in part might be due to the different criteria used to diagnose IBS (i.e., Rome II or Rome III Criteria) and also to the different spectrum of patients included (i.e., diverticulosis, SUDD or patients with previous diverticulitis, whether considered separately or not) [4, 6, 7, 13, 16, 18]. Regarding the IBS subtype, we found that IBS-D is the most common subtype, similarly to what previously reported [28]. In fact, a large population-based cross-sectional survey conducted by mailing and using Rome II Criteria, found that the presence of IBS was associated to DD (OR 1.7, 95% CI 1.2–2.4), especially the IBS-D (OR 1.9, 95% CI 1.1–3.2) [28].

The biological mechanisms that link DD and IBS-like symptoms are still matter of debate. It has been reported that alterations of intestinal microbiota, low grade inflammation and nerve sprouting may have a role in diverticular disease as in IBS [29–31]. In fact, as reported by Barbara et al. patients with colonic diverticular disease show depletion of microbiota members with anti-inflammatory activity (Clostridium cluster IV, Clostridium cluster IX, Fusobacterium and Lactobacillaceae) associated with mucosal macrophage infiltration [32]. Dysbiosis has often been suggest as an important player in SUDD pathogenesis and in symptoms generation [29]. Tursi et al. compared fecal microbiota of SUDD patients with both asymptomatic diverticulosis patients and healthy controls, showing a different composition of gut microbiota in diverticular disease patients compared to healthy controls [33]. Also, Kvasnovsky et al. showed that abdominal symptoms in SUDD patients might correlate with alterations in fecal microbiota, such as the severity of abdominal bloating which appears to associated with higher levels of bacteria involved in hydrogen production [34]. However, even if dysbiosis has been suggested as an important player in DD pathogenesis, only few and heterogenous studies are available and needed to be confirmed in further larger studies.

This study has some limitations. The main limit is the cross-sectional design, which does not allow to establish whether symptoms compatible with IBS were present before the detection of diverticula or not. In addition, the study has been designed in 2015, including Rome III instead of Rome IV criteria. In Rome III, a diagnosis of IBS included chronic abdominal pain or discomfort at least 3 days per month whereas in Rome IV the term discomfort has been removed and the pain frequency increased to at least 1 day per week [35]. This could lead to a possible overestimation of IBS diagnosis. Nevertheless, this is the first study that systematically assessed clinical features of abdominal pain, in terms of presence, severity and length, in patients with SUDD and PD using standardized questionnaires for DD and IBS in a large multicentric cohort.

We found that SUDD and PD patients presented different pattern of abdominal pain (i.e., pain’s length, number of long lasting pain episodes, localization and associated features), with only a third of the study population reporting an overlap with IBS. Further observational studies are needed to better characterize abdominal symptoms in patients with different spectrum of diverticular disease, especially in patients not fulfilling IBS diagnosis.

### Supplementary Information

Below is the link to the electronic supplementary material.

Supplementary file1 (PDF 89 KB)

## Data Availability

Data supporting this study are available from the corresponding author upon reasonable request.
